# Plant-based zinc nanoflowers assisted molecularly imprinted polymer for the design of an electrochemical sensor for selective determination of abrocitinib

**DOI:** 10.1007/s00604-024-06404-2

**Published:** 2024-05-10

**Authors:** Ahmet Cetinkaya, Sadi Yusufbeyoglu, S. Irem Kaya, Ayse Baldemir Kilic, Esen Bellur Atici, Sibel A. Ozkan

**Affiliations:** 1 Department of Analytical Chemistry, Faculty of Pharmacy, Ankara University, Ankara, 06560 Turkey; 2 Department of Pharmaceutical Botany, Gulhane Faculty of Pharmacy, University of Health Sciences, Ankara, Turkey; 3 Department of Analytical Chemistry, Gulhane Faculty of Pharmacy, University of Health Sciences, Ankara, Turkey; 4 DEVA Holding A.S., Research&Development Center, Tekirdag, 59510 Turkey

**Keywords:** Abrocitinib, Zn-Nanoflower, *Alkanna cappadocica*, Molecularly imprinted polymer, Graphene oxide; Modified glassy carbon electrode, Differential pulse voltammetry

## Abstract

The first electrochemical sensor application in the literature is described for the sensitive and selective determination of the selective Janus kinase (JAK)-1 inhibitor abrocitinib (ABR). ABR is approved by the U.S. Food and Drug Administration (FDA) for the treatment of atopic dermatitis. The molecularly imprinted polymer (MIP)-based sensor was designed to incorporate zinc nanoflower (ZnNFs)-graphene oxide (GO) conjugate (ZnNFs@GO), synthesized from the root methanolic extract (RME) of the species *Alkanna cappadocica* Boiss. et Bal. to improve the porosity and effective surface area of the glassy carbon electrode (GCE). Furthermore, the MIP structure was prepared using ABR as a template molecule, 4-aminobenzoic acid (4-ABA) as a functional monomer, and other additional components. Scanning electron microscopy (SEM), X-ray diffraction (XRD), and Fourier transform infrared spectroscopy (FTIR) were used to characterize the surface and structure of the synthesized nanomaterial and MIP-based surface. Among the electrochemical methods, cyclic voltammetry (CV) and electrochemical impedance spectroscopy (EIS) were preferred for detailed electrochemical characterization, and differential pulse voltammetry (DPV) was preferred for all other electrochemical measurements using 5.0 mM [Fe(CN)_6_]^3–/4–^ solution as the redox probe. The MIP-based sensor, which was the result of a detailed optimization phase, gave a linear response in the 1.0 × 10^–13^ – 1.0 × 10^–12^ M range in standard solution and serum sample. The obtained limit of detection (LOD) and limit of quantification (LOQ) values and recovery studies demonstrated the sensitivity, accuracy, and applicability of the sensor. Selectivity, the most important feature of the MIP-based sensor, was verified by imprinting factor calculations using ibrutinib, ruxolitinib, tofacitinib, zonisamide, and acetazolamide.

**Graphical Abstract:**

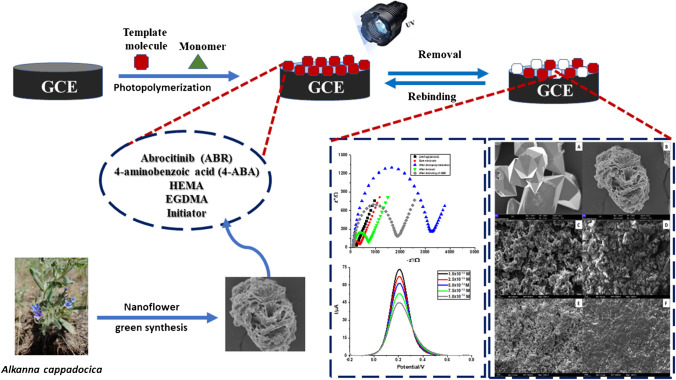

## Introduction

Atopic dermatitis, more commonly known as eczema, is a skin disease with an inflammatory and chronic profile. Patients with atopic dermatitis may experience physical and psychological symptoms, including itching and pain, as well as anxiety and depression. The onset of the disease can begin as early as infancy and, in some patients, can continue into adulthood. There is a need to find effective treatments for atopic dermatitis, which has a significant impact on patients’ quality of life. The long treatment process requires reliable medications with high benefits [1–3]. Current treatment options include several topical and systemic approaches. Janus kinase (JAK) inhibition has gained importance as a novel and effective alternative in systemic treatment [1]. Abrocitinib (ABR) selectively inhibits JAK-1, thereby inhibiting signaling in pathways associated with the pathogenesis of atopic dermatitis. In addition, JAK1-specific inhibition eliminates the risk of undesirable side effects such as neutropenia and anemia [4]. ABR has been approved by the U.S. Food and Drug Administration (FDA) for the treatment of moderate and severe atopic dermatitis. The commonly prescribed daily dose is 100 to 200 mg [2, 4, 5]. It is essential to determine ABR in biological fluids for clinical follow-up and monitoring of its side effects and possible toxic effects because it is still a novel drug and its approved use is relatively new.

There is only one analytical method reported in the literature for the determination of ABR from plasma with other JAK inhibitors (tofacitinib, ruxolitinib, etc.) using high-performance liquid chromatography coupled to tandem mass spectrometry (HPLC–MS/MS) [6]. In addition, studies in the literature have focused on elucidating the pharmacokinetic profile of ABR [4, 7, 8]. In light of this information, it seems that reliable, cost-effective, sensitive, and rapid methods are needed for the determination of ABR. Electrochemical methods have long been preferred as easy-to-use, affordable, rapid, highly sensitive, and reliable options for the determination of a wide variety of analytes in different samples, such as biological, pharmaceutical, and environmental samples. Despite all these advantages, the selectivity problem limits the use of electrochemical sensors. As a solution, it is preferred to integrate molecularly imprinted polymers (MIPs) into electrochemical sensors, which provide selectivity thanks to recognition regions explicitly designed for the target molecule in their structure. In addition to selectivity, the high stability, low cost, and relatively simple preparation procedure of MIPs are outstanding advantages. Moreover, the incorporation of various nanomaterials into the MIP design improves the electrochemical properties such as surface porosity and surface area [9–12].

Nanotechnology is being developed in many fields. Nanoparticles are widely used as catalysts, medical imaging, and drug delivery systems, especially in biotechnology, due to their unique shapes, properties, and application areas. Recently, hybrid functional molecules formed by the interaction of metal ions and organic/biomolecules have attracted great interest due to their high activity relative to their components. Flower-like nanohybrid structures have been synthesized using organic molecules and metal ions as inorganic components, and their various activities have been investigated. Although nanoparticles have cost-effective synthesis conditions, the use of toxic chemicals and solvents limits medical and clinical applications. Current studies mainly focus on the synthesis of metallic nanoparticles using non-toxic and biocompatible materials. These synthesis methods are classified as green synthesis [13]. Green synthesis aims to reduce the hazardous and toxic substances used in the production of nanoparticles and replace them with non-toxic substances. The use of plants and plant extracts in this synthesis method has attracted the attention of researchers [14]. In recent studies, nanoflower structures have been synthesized using plants, plant extracts, and active components and have been used in many biological activity studies and sensor applications [15–18]. Organic–inorganic synthesized nanoflowers are commonly included in electrode sensors to enhance their sensitivity by increasing the surface area of modified electrodes. Zinc (Zn) nanoparticles are commonly selected for the fabrication of efficient amperometric sensors due to their biocompatibility, non-toxicity, chemical stability, large surface area, and electrical conductivity [19, 20].

In this study, it was aimed to improve the electrochemical performance of the MIP structure by increasing the porosity and surface area by incorporating zinc nanoflower (ZnNFs)-graphene oxide (GO) conjugate (ZnNFs@GO) synthesized from the root methanolic extract of *Alkanna cappadocica* plant. An electrochemical sensor (ABR/4-ABA/ZnNFs@GO/MIP/GCE) using ZnNFs@GO-assisted MIP was designed for the highly sensitive and selective determination of ABR. In designing the MIP in the presence of ZnNFs@GO, 4-aminobenzoic acid (4-ABA) was chosen as the functional monomer, and the complementary components were the basic monomer 2-hydroxyethyl methacrylate (HEMA), the crosslinker ethylene glycol dimethacrylate (EGDMA), and the photoinitiator 2-hydroxy-2-methylpropiophenone. Photopolymerization was used as the polymerization technique in the sensor developed on the glassy carbon electrode (GCE) surface, and the resulting sensor (ABR/4-ABA/ZnNFs@GO/MIP/GCE) was applied to both standard solution and commercial serum sample. This study has the significant feature of being the first electrochemical sensor with high sensitivity and selectivity for ABR determination in the literature.

## Experimental

### Plant material

The root parts of *Alkanna cappadocica* were collected in November from the Harman region of Ali Dağ, Kayseri, Türkiye, at an altitude of 1268 – 1442 m (38° 40.25′ N, 35° 32.18′ E). The plants were dried in a cool and dark place. The herbarium specimens are stored in the Herbarium of the Faculty of Pharmacy at Ankara University (AEF-30976).

### Chemicals and reagents

Abrocitinib (ABR, ≥ 99.5%), ruxolitinib (≥ 99.5%), ibrutinib (≥ 99.5%), tofacitinib (≥ 99.5%), zonisamide (≥ 99.5%), and acetazolamide (≥ 99.5%) were provided by DEVA Holding A.S. (Istanbul, Türkiye). Stock solutions of the drug substances were prepared using methanol (≥ 99.8%) from Sigma-Aldrich. Other chemicals purchased from Sigma-Aldrich were *N*,*N*-dimethylformamide (DMF, ≥ 99.0%) used for the dispersion of nanomaterials; hydrochloric acid (HCl, ≥ 37.0%), sodium hydroxide (NaOH, ≥ 97.0%), acetic acid (HAc, ≥ 99.0%), acetonitrile (ACN, ≥ 99.9%), and acetone (≥ 99.0) used for the removal step; HEMA (≥ 99.9%), EGDMA (> 98.0%), and 2-hydroxy-2-methyl propiophenone (≥ 97%) used for the MIP sensor preparation; potassium chloride (KCl, ≥ 99.0%) used for the redox probe preparation; potassium nitrate (≥ 99.0%), magnesium chloride (≥ 99.0%), sodium sulfate (≥ 99.0%), dopamine (≥ 97.5%), ascorbic acid (≥ 99.0%), uric acid (≥ 99.0%), and paracetamol (≥ 99.0%) used for the interference studies. In addition, the following chemicals were purchased from Merck: Potassium ferricyanide (K_3_[Fe(CN)_6_], ≥ 98.5%) and potassium ferrocyanide (K_4_[Fe(CN)_6_]0.3H_2_O, ≥ 99.0%) used for the preparation of the redox probe; 4-aminobenzoic acid (4-ABA, ≥ 99.0%) used as the functional monomer; copper (II) sulfate (CuSO_4_, ≥ 99.0%), sodium chloride (NaCl, ≥ 99.0%), disodium hydrogen phosphate (Na_2_HPO_4_, ≥ 99.0%), and potassium dihydrogen phosphate (KH_2_PO_4_, ≥ 99.0%) used for nanomaterial preparation.

### Apparatus

AUTOLAB (Metrohm AG, Switzerland) with NOVA 2.1.5 software was used as a potentiostat for electrochemical measurements, including CV, DPV, and EIS. In addition to the potentiostat, a working electrode consisting of a glassy carbon electrode (GCE, diameter: 3.0 mm), a reference electrode consisting of an Ag/AgCl electrode in 3 M KCl, and a counter electrode consisting of a platinum wire electrode were used to complete the electrochemical measurement system. All the above electrodes were purchased from BASi (USA). The precision balance (Ohaus Instruments, Shanghai, China) is used to weigh the solid chemicals. The vortex mixer (ISOLAB Laborgerate GmbH, Germany) and ultrasonic bath (J.P. Selecta Co., Barcelona, Spain) were used in the solution preparation phase. A UV lamp (100 W, Analytik Jena US LLC, Canada) with a wavelength of 365 nm was used for photopolymerization in the MIP preparation procedure. Removal and rebinding steps were performed using a ThermoShaker (Biosan TS-100, Riga, Latvia). A scanning electron microscope (SEM, TESCAN GAIA 3, Brno-Kohoutovice, Czech Republic) was used for the surface characterization. The crystal structures of NFs were determined by X-ray diffraction (XRD, Malvern Panalytical, Empyrean) and chemical structures by Fourier transform infrared spectroscopy (FTIR, Perkin Elmer 400 FT-IR Spectrometer Spotlight 400 Imaging System). A rotary evaporator (Buchi R215) was used to evaporate methanol from the extract and Lyoflizer (Lyof-christ gamma 2–20) was used to obtain powder extract.

### Preparation of *Alkanna cappadocica* root extracts

The roots of *A. cappadocica* were crushed with a mechanical grinder and 100 g of the sample was weighed. The root extracts were prepared by the maceration method with methanol at room temperature in the dark for 24 h. After maceration, the liquid extract was obtained by filtration with filter paper (Whatmann No: 1). The solvents were evaporated with rotavapor and then lyophilized to obtain dry extracts. It was stored at –20 °C until the time of the experiment.

### Green synthesis of ZnNFs and ZnNFs@GO

First, 120 mM zinc acetate (Zn(CH_3_CO_2_)_2_) stock solution is prepared in distilled water. A certain volume of zinc acetate solution is added to phosphate buffer (PBS, 10 mM, pH: 7.4) containing 0.02 mg/mL plant extract, and the mixture is rapidly stirred for about 30 s (Zn^+2^ concentration in the final mixture should be 0.8 mM). The mixture is then incubated for 72 h without intervention. The reaction is then stopped by centrifugation and the resulting blue or colored precipitate is characterized by drying the NFs [21, 22]. Then, 1 mg of graphene oxide (GO) was dispersed in 1 mL of distilled water, and varying amounts of ZnNFs (1 mg, 2 mg, and 3 mg) were added and incubated overnight on the vortex at room temperature. After incubation, excess GO was removed by centrifugation at 5000 rpm, dispersed in 1 mL of distilled water, and stored for further experiments [23]. In addition, the obtained *A. cappadocica* root extract was prepared at 10 mg/mL; later, 2 mL of extract and 50 mM HCl with 5 mL of AgNO_3_ for synthesized silver nanoparticles (AgNPs) using the method of Zheng et al. (2015) [24, 25].

### Fabrication of the ZnNFs@GO-assisted MIP-based sensor

A detailed cleaning step is required to prepare the electrode surface for MIP film formation. A mixture of equal volumes of distilled water and methanol is prepared in which the GCE is immersed and placed in an ultrasonic bath for 15 min. In the polishing step, the GCE surface is again cleaned using an alumina slurry and a polishing pad. After rinsing with distilled water, the electrode is dried at room temperature. Once the electrode surface is ready for polymerization, the polymerization solution is prepared. First, 2 mg of zinc nanoflowers synthesized from root methanolic extract (ZnNFs)-graphene oxide (GO) conjugate (ZnNFs@GO) are dispersed in 1 mL DMF and kept in an ultrasonic bath for 2 h. Twenty microliters of ZnNFs@GO dispersion is taken, combined with 20 µL template molecule ABR (1.0 mM) and 20 µL functional monomer 4-ABA (1.0 mM) in an Eppendorf tube and mixed by vortex mixing for 30 s. To this mixture, 100 µL of basic monomer HEMA and 20 µL of crosslinker EGDMA are added and mixed again by vortex mixing for 30 s. Finally, 20 µL of this mixture is transferred to a separate Eppendorf tube, 2 µL of the photoinitiator 2-hydroxy-2-methylpropiophenone is added, and the mixture is mixed again for 30 s. The optimized amount of 0.25 µL of this photoinitiator-containing mixture is taken and dropped onto the GCE surface. To initiate photopolymerization, the GCE is exposed to 365 nm UV light for 10 min. A MIP film is then formed on the surface of the electrode, which is held at room temperature for 10 min (ABR/4-ABA/ZnNFs@GO/MIP/GCE). The ABR-specific cavities in the ABR/4-ABA/ZnNFs@GO/MIP/GCE sensor structure are revealed by a removal process. The GCE is immersed in 15 M HAc solution in an Eppendorf tube and kept on a ThermoShaker at 650 rpm for 10 min. Rebinding is performed using the same technique but using ABR solutions at the desired concentrations for 10 min instead of a removal solution. If all of the above steps are followed exactly, but prepared without adding ABR to the polymerization solution, a non-imprinted polymer (NIP) is obtained that does not have specific cavities. With NIP/GCE, the performance and selectivity of the ABR/4-ABA/ZnNFs@GO/MIP/GCE sensor are tested at each step. The indirect measurement technique is used for all electrochemical measurements with the redox probe 5.0 mM [Fe(CN)_6_]^3–/4–^ solution.

### Preparation of commercial samples of human serum

The accuracy and feasibility of the ABR/4-ABA/ZnNFs@GO/MIP/GCE sensor were evaluated using a commercial sample of human serum. For sample preparation, the commercial serum was removed from the –20 °C freezer and allowed to thaw at room temperature. To prepare the serum stock solution, 1.8 mL of serum at room temperature was placed in a centrifuge tube and mixed with 2.7 mL of acetonitrile and 0.5 mL of 1.0 mM ABR. After 20 min of centrifugation (5000 rpm), the serum proteins are separated by precipitation and the supernatant is the stock serum solution. Necessary dilutions are prepared from the stock serum solution and used to evaluate analytical performance. In addition, a recovery study is performed by spiking ABR at a known concentration to evaluate accuracy.

### Characterization studies

Scanning electron microscopy (SEM) was used to image the NFs and sensor surfaces, X-ray diffraction (XRD) was used to investigate the crystalline structures formed, Fourier transform infrared spectroscopy (FTIR) was used to characterize and reveal the chemical structures, and energy dispersive X-ray spectrometry (EDX) was used to show the presence of zinc in the NFs.

## Results and discussion

### Surface characterization of ABR/4-ABA/ZnNFs@GO/MIP/GCE sensor

**Fig. 1:**
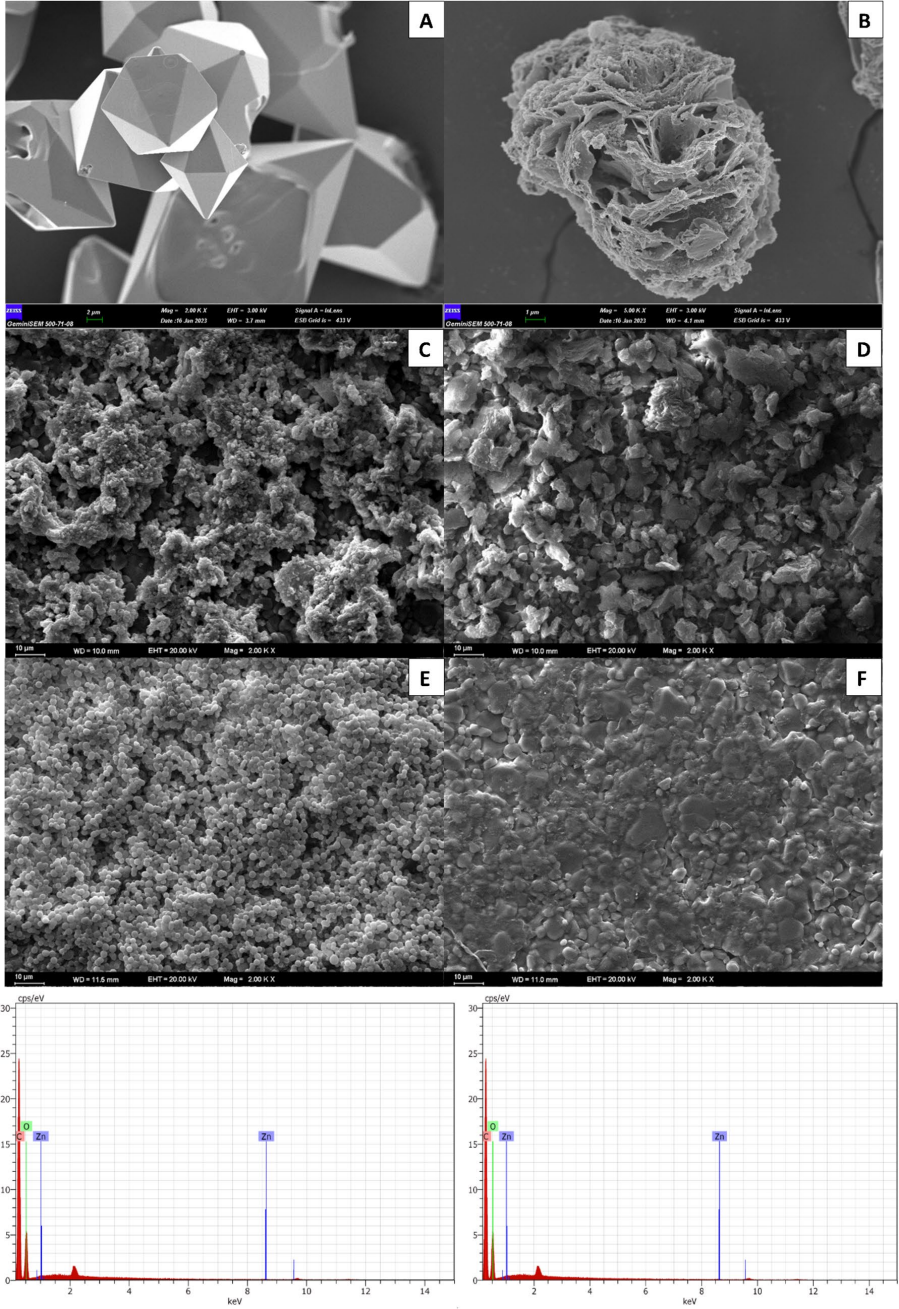
SEM images of **A** ZPO, **B** ZnNFs, **C** ZnNfs@GO, **D** GO, **E** ABR/4-ABA/ZnNFs@GO/MIP/GCE, and **F** 4-ABA/ZnNFs@GO/NIP/GCE; the EDX spectra of **G** ABA/ZnNFs@GO/MIP/GCE and **H** ABA/ZnNFs@GO/NIP/GCE

The morphological analysis of the ZnNFs, GO, ZnNfs@GO, and polymer structures synthesized on the electrode in this study was performed by scanning electron microscopy (SEM). The SEM images shown in Fig. 1 indicate that the synthesized nanomaterials and sensor surfaces have a porous structure. When the images were examined, it was found that zinc phosphate (ZPO) crystals (Fig. 1A) and ZnNFs (Fig. 1B) with a porous structure were successfully synthesized. In addition, more porous materials were obtained by combining ZnNFs with GO structures (Fig. 1D). ZnNFs@GO (Fig. 1C) is believed to provide an advantage to the material to be degraded by increasing the distribution of these pore structures. It can also be seen that there is a significant morphological difference between the obtained MIP (Fig. 1E) and NIP (Fig. 1F). In the MIP structure (Fig. 1E), it can be seen that the ZnNFs are bound according to the template, and due to the removal of this template, it has more pores and heterogeneous distribution compared to the NIP (Fig. 1F). The porous morphology leads to easy accessibility of the template to the recognition sites. The surface characterization of ABR/4-ABA/ZnNFs@GO/MIP/GCE was also performed by energy dispersive X-ray spectroscopy (EDX) analysis, and the EDX spectra of the ABR/4-ABA/ZnNFs@GO/MIP/GCE sensor showed that the polymer matrix contains C, O, Zn, and P atoms in its structure (Fig. 1G and H). EDX analysis also shows that this sensor contains zinc nanoflower structures.

**Fig. 2:**
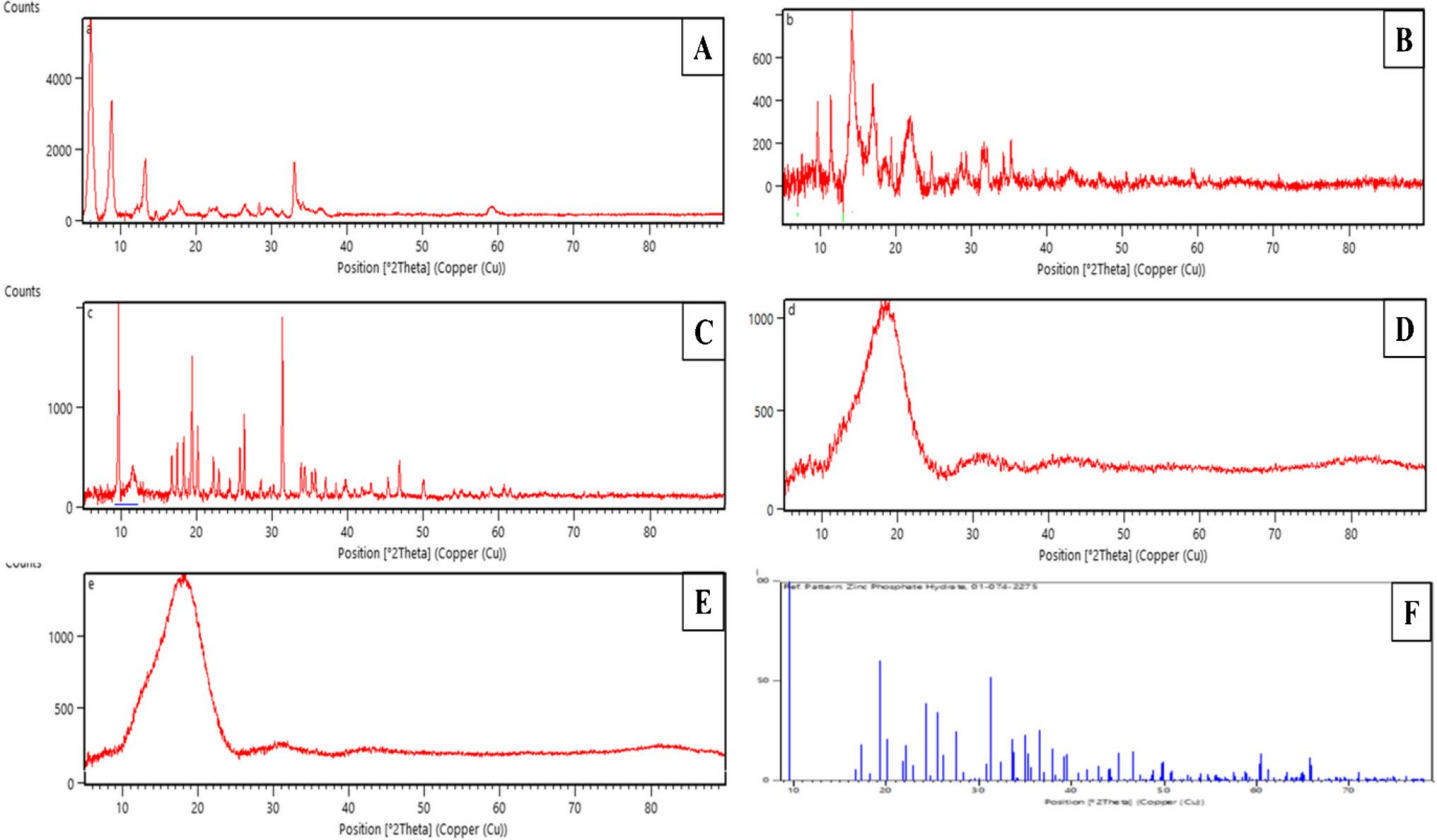
XRD analysis of **A** ZPO, **B** ZnNFs, **C** ZnNFs@GO, **D** ABR/4-ABA/ZnNFs@GO/MIP/GCE, **E** 4-ABA/ZnNFs@GO/NIP/GCE, and **F** Zn(PO)_4_ crystals

XRD analysis was performed to show ZPO in the structure (Fig. 2A) of the formed ZnNFs and ZnNFs@GO (Fig. 2B and C). A similar pattern was obtained with the standard Zn_3_(PO_4_)_2_.4H_2_O (ZPO, JCPDS card no. 00–001-0975, Fig. 2F). The XRD spectra of the phosphate crystals obtained in Fig. 2B and C were also observed within the spectrum of ZnNFs and ZnNFs@GO structures. The MIPs (Fig. 2D) and NIPs (Fig. 2E) were similar with a broad hump around 2*θ* = 18.17°. It corresponds to the C(002) plane, which explains its amorphous structure. Another small peak appeared at 44.65° associated with the C(100) plane, representing the graphitic carbon spacing [26, 27].

**Fig. 3:**
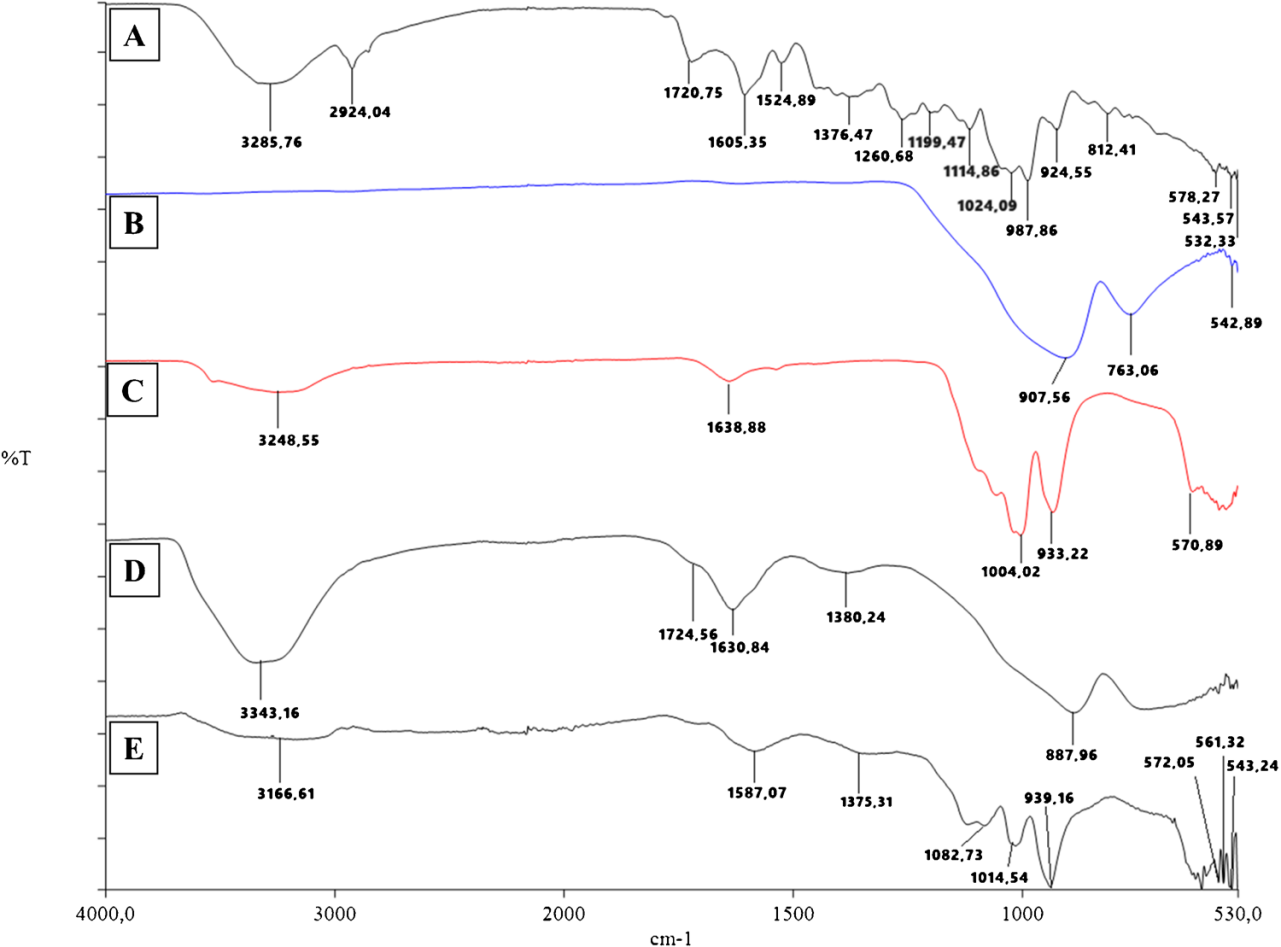
FTIR spectra of **A** *A. cappadocica* root extract, **B** ZPO, **C** ZnNFs, **D** GO, and **E** NFs@GO

In order to verify the functional groups of the synthesized material, the chemical characterization of the developed sensor was investigated using ATR-FTIR spectra measured in the range of 4000 – 650 cm^–1^. Through FTIR analysis, the interatomic bonds of the extracts, the content of the synthesized NCs and polymeric sensor, the bonding sites of the atoms, and the aromatic or aliphatic structure of the structure were investigated whether it contains a phosphate structure. The FTIR spectra of the extract, ZPO, ZnNFs, GO, and ZnNfs@GO are shown in Fig. 3. When the bands in the FTIR spectrum of the root extract (Fig. 3A) are examined, the broad peak ~ 3286 cm^–1^ O–H or N–H stretching, ~ 2924 cm^–1^ aliphatic C–H stretching, ~ 1721 cm^–1^ C = O stretching, ~ 1525 cm^–1^ and ~ 1605 cm^–1^ C = C stretching, ~ 1377 cm^–1^ C–O stretching, 1261 – 1024 cm^–1^ C heteroatom single bond stretching, ~ 1119 cm^–1^, ~ 1115 cm^–1^ C–C stretching, ~ 988 cm^–1^ and ~ 925 cm^–1^ C–H bending vibrations are shown. FTIR analysis of the ZPO structure was performed to observe the (PO_4_)_3_ groups contained in the NFs. The bending in the vibrational bands of primary phosphate crystals was observed at 908 – 543 cm^–1^ [28, 29] (Fig. 3B). The bending in the vibrational bands of (PO_4_)_3_ groups includes extract ZnNFs 939 – 571 cm^–1^. In addition, O–H / N–H, C–O, and C–C stretching originating from the extract were observed. ZnNFs show similarity with the ZPO FTIR spectrum [30, 31]. The bands mentioned in the extract were not observed in ZnNFs. This suggests that these bands cannot be observed due to their encapsulation in the formation of ZnNFs (Fig. 3C).

The absorption peaks at ~ 3343 cm^–1^ correspond to O–H stretching, ~ 1725 cm^–1^ to C = O stretching of the carboxyl group, ~ 1631 cm^–1^ to C = C stretching, and ~ 1380 cm^–1^ to C–O stretching of the alcohol groups on the GO structure (Fig. 3D). In the ZnNFs@GO structure, it can be seen that the hydroxyl groups are encapsulated in the structure, and the peak shifts to ~ 3167 cm^–1^, and the ZPO structure bending in the vibrational bands of primary phosphate crystals was observed at ~ 908 – 572 cm^–1^. At the same time, functional groups from extract and GO were detected in ZnNFs (Fig. 3E).

**Fig. 4:**
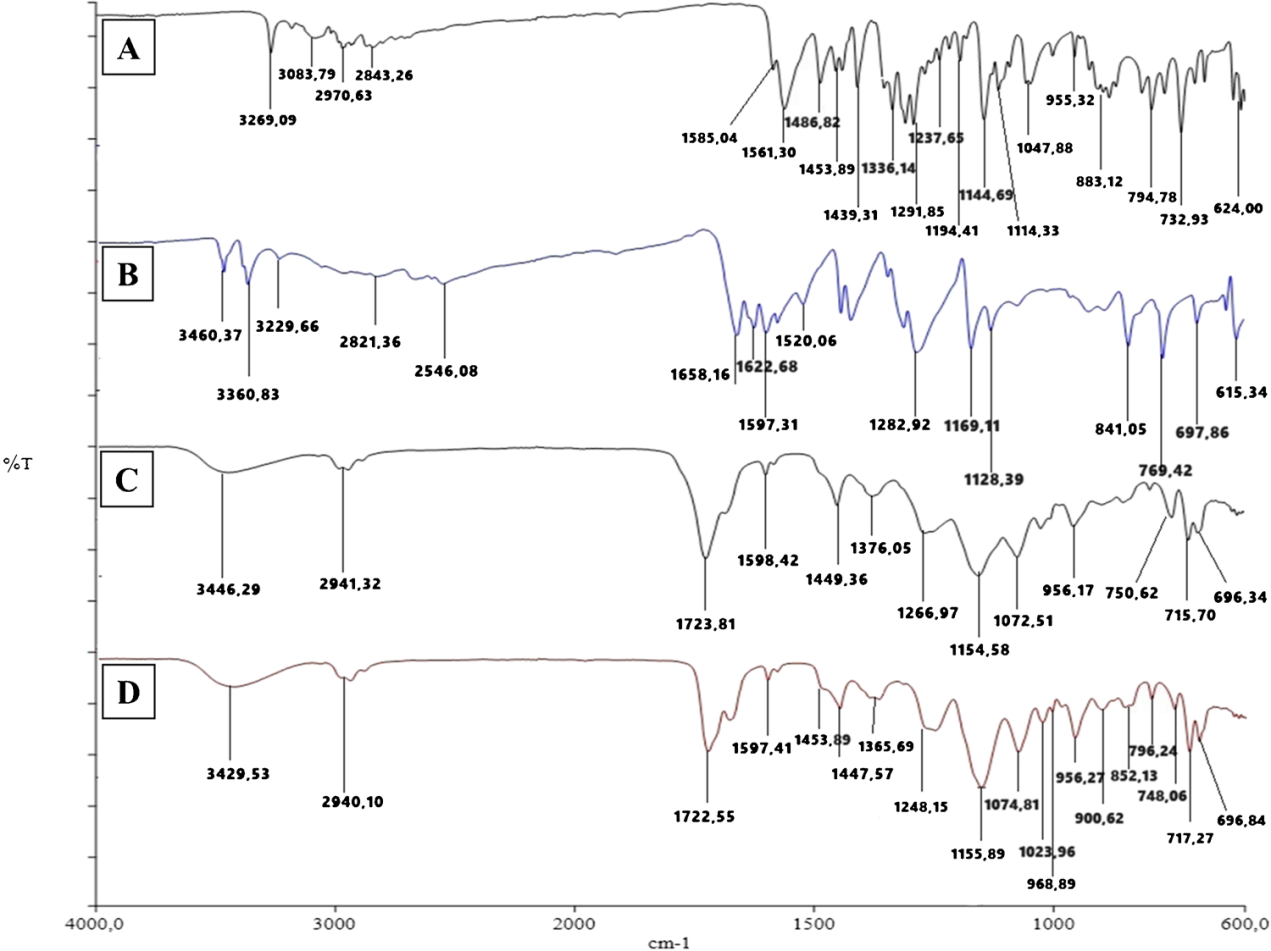
FTIR spectra of **A** ABR, **B** 4-ABA, **C**4-ABA/ZnNFs@GO/NIP/GCE, and **D** ABR/4-ABA/ZnNFs@GO/MIP/GCE

Figure 4A shows the FTIR spectrum of ABR and the peaks were observed at ~ 3269 cm^–1^ for N–H, ~ 3084 cm^–1^ for aromatic C–H, ~ 1584 cm^–1^ and ~ 1561 cm^–1^ for C = C / C = N, ~ 1292 cm^–1^ for aromatic amine, ~ 1194 cm^–1^ for C–N stretching of amine peak, and O = S = O / S = O stretching at ~ 1145 cm^–1^, ~ 1114 cm^–1^ and ~ 1048 cm^–1^. FTIR of 4-ABA (Fig. 4B) shows the presence of N–H at ~ 3461 cm^–1^ and ~ 3360 cm^–1^, O–H at ~ 3230 cm^–1^, aromatic C–H at ~ 2821 cm^–1^ and ~ 2546 cm^–1^, C = O at ~ 1658 cm^–1^ and aromatic C–N at ~ 1283 cm^–1^. The FTIR spectra of NIP (Fig. 4C) and MIP (Fig. 4D) were similar and the C = O peaks at ~ 1724 cm^–1^ and ~ 1723 cm^–1^ are from the HEMA structure [28, 29]. Due to polymerization, the N–H and O–H of 4-ABA are closed. The ABR peaks were also not clearly observed in Fig. 4D. However, the peaks at ~ 1156 cm^–1^, ~ 1075 cm^–1^, and ~ 1024 cm^–1^ belonging to O = S = O and S = O bonds were observed differently due to the ABR content in MIP, and an increase in the peak intensities in this region was observed in Fig. 4D.

### CV and EIS methods-based characterization of ABR/4-ABA/ZnNFs@GO/MIP/GCE sensor

**Fig. 5:**
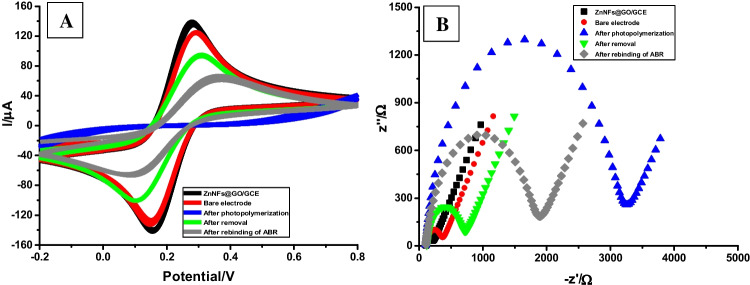
**A** CV voltammograms and **B** EIS Nyquist plots belonging to the redox probe for the different stages of the ABR/4-ABA/ZnNFs@GO/MIP/GCE sensor preparation. (Conditions for CV: potential scan range: − 0.2 V to + 0.8 V, scan rate: 0.05 V/s, step potential: 0.01 V; for EIS: minimum frequency: 0.1 Hz, maximum frequency: 100,000 Hz, Eac: 0.01 V)

The interactions and surface changes during the MIP formation, removal, and rebinding processes on the GCE surface also affect some electrochemical properties. The most important properties are conductivity, electron transfer rate, and charge transfer resistance (*R*_ct_). CV and EIS methods are used to observe and follow the changes in these properties. Thanks to CV measurements, the conductivity and electron transfer rate on the GCE surface are evaluated by the increase/decrease of the oxidation–reduction peaks of the redox probe. As shown in Fig. 5A, CV measurements were performed on bare GCE, ZnNFs@GO modified GCE, after photopolymerization, removal, and rebinding of ABR steps. Due to the surface area, conductivity, and electron transfer enhancing effects of ZnNFs@GO, the peak of the redox probe increased compared to the bare GCE. From now on, when photopolymerization occurs, it should be observed that the MIP film on the surface blocks the electron transfer. Therefore, the oxidoreduction behavior of the redox probe can hardly be observed. As mentioned above, the purpose of the removal step is to remove the template molecule from the polymer structure and create cavities with specific binding sites for the target molecule. The formation of these cavities allows electron transfer to be activated on the GCE surface. In this case, the peaks of the redox probe increase. The rebinding step is the step where the electron transfer on the surface is partially blocked by the binding of ABR to the cavities. For this reason, it is observed that the peak current of the redox probe decreases. Another electrochemical property of the redox probe is the *R*_ct_ value. For this purpose, Nyquist plots obtained with EIS measurements are evaluated at the same stages as CV (Fig. 5B). However, as electron transfer becomes easier, the CV peak current values increase while the *R*_ct_ values decrease in the EIS method. In other words, there is an opposite logic to the events observed in CV. The *R*_ct_ values obtained are shown in Table 1.

**Table 1:** Rct values obtained for ABR/4-ABA/ZnNFs@GO/MIP/GCE sensor preparation

	*R*_ct_ value (Ω)
ZnNFs@GO/GCE	102
Bare GCE	136
After photopolymerization	3020
After removal	564
After rebinding of ABR	1700

### Critical parameter optimization affecting MIP sensor performance

#### Nanomaterial type and amount

**Fig. 6:**
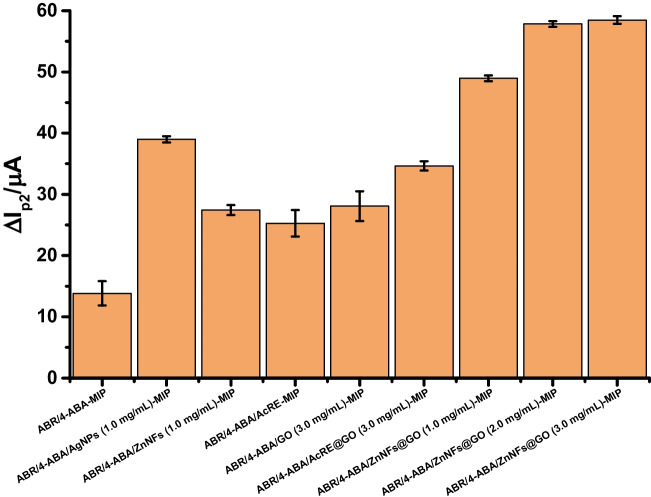
The plot of Δ*I*_2_ values versus different nanomaterials used to prepare MIP-based sensor

In electrochemical sensors, increasing the conductivity and electron transfer rate of GCE improves the performance of the sensor. Various nanomaterials can be used for this purpose. Due to the advantageous properties of nanomaterials, such as good electrical conductivity and large surface area, it is possible to enhance electrochemical processes [9, 32]. By observing the effect of adding nanomaterials to the MIP structure on the Δ*I*_2_ values (the difference between the peak current values obtained after removal and rebinding), it was aimed to select the most suitable nanomaterial to improve the MIP performance. For this purpose, *Alkanna cappadocica* root extract (AcRE), silver nanoparticles (AgNPs), and zinc nanoflowers (ZnNFs) synthesized from the root extract and their graphene oxide (GO) conjugate (ZnNFs@GO) were tested. Since the best result was obtained with ZnNFs@GO, the effect of different concentrations was also evaluated. Since very similar results were found at 2 mg/mL and 3 mg/mL concentrations, the MIP sensor was prepared using 2 mg/mL ZnNFs@GO in the next steps (Fig. 6).

#### Functional monomer-to-template ratio

**Fig. 7:**
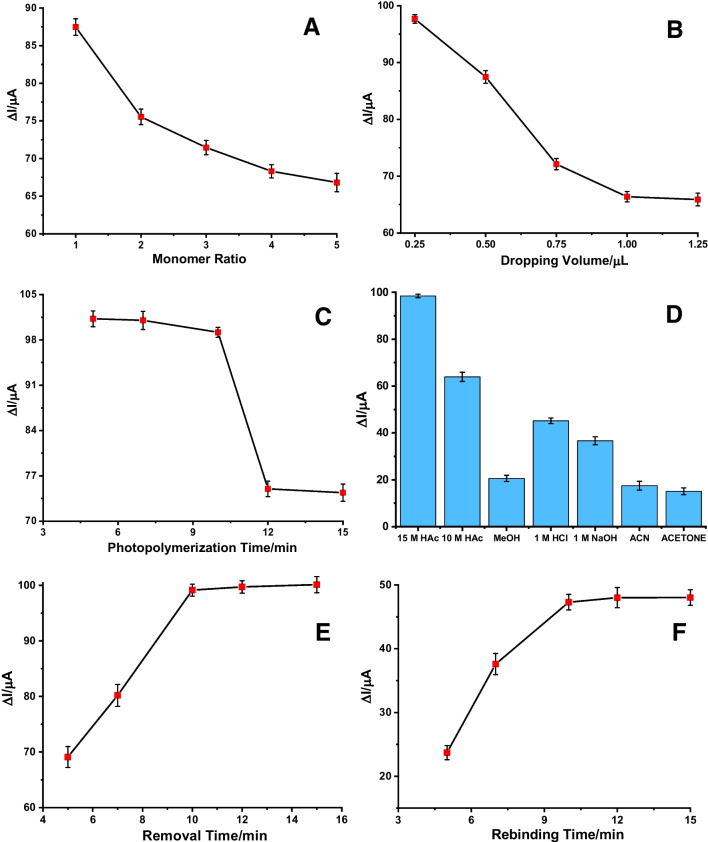
The plot of ΔI_1_ values versus **A** functional monomer to template ratio, **B** dropping volume, **C** photopolymerization time, **D** removal solutions, **E** removal time, and Δ*I*_2_ values versus **F** rebinding time for ABR/4-ABA/ZnNFs@GO/MIP/GCE sensor (each value is the mean of three experiments.) (Conditions for DPV: potential scan range, − 0.2 V to + 0.6 V; scan rate, 0.001587 V/s; step potential 8 mV; modulation amplitude 50 mV; modulation time 0.05 s, interval time 0.5 s)

The basis of the polymeric structure of the MIP is the primary interaction between the template molecule and the functional monomer. For the most efficient interaction, the ratio of functional monomer to template must be properly adjusted. If the amount of functional monomer is less than optimal, the functional groups cannot effectively participate in the resulting polymeric structure. This leads to problems with the creation of fewer selective cavities and lower porosity. In cases where the amount of functional monomer is more than optimal, the monomers may be in random locations in the polymeric structure instead of forming selective cavities. Therefore, for each ratio, the Δ*I*_1_ values, which are the difference between the peak currents obtained after removal and after photopolymerization, are examined. As shown in Fig. 7A, the highest ΔI_1_ value belongs to the 1:1 ratio, and then a gradual decrease is observed. According to this graph, which indicates that the 1:1 ratio is optimally selected, the removal efficiency decreases with increasing functional monomer ratios.

#### Dropping volume

It is also necessary to optimize how much of the photopolymerization solution, prepared according to the optimal functional monomer to template ratio, is taken up and applied to the GCE surface in the final step. The drop volume in this step is also relevant to other optimization steps because it is expected to affect the MIP film thickness and the photopolymerization time. Figure 7B shows the relationship between Δ*I*_1_ values and drop volumes from 0.25 to 1.25 µL. Accordingly, almost the same values were obtained at 1 µL and 1.25 µL, with a decrease observed after the highest value was obtained at 0.25 µL. Therefore, the drop volume was determined to be 0.25 µL. As the volume increases, a very strong polymer is formed on the surface after polymerization, which makes the removal step difficult.

#### Photopolymerization time

The photopolymerization time directly affects the total time required to design and prepare the sensor for measurement. This refers to how long UV light is applied after the optimal amount of photopolymerization solution is dropped onto the GCE surface. Figure 7C shows that polymerization times of 5, 7, 10, 12, and 15 min were examined against Δ*I*_1_ values. Accordingly, the highest and very similar results were obtained at 5, 7, and 10 min, with a decrease at 12 min. However, the most stable and repeatable results were obtained at 10 min, which was selected as the optimal photopolymerization time. After exposure to UV light, GCE was held at room temperature for another 10 min.

#### Removal solution and time

Selecting the appropriate solution and duration of the removal phase is one of the most critical optimization steps. In the removal step, the template molecule is removed from the MIP structure, creating ABR-specific cavities. While forming the MIP structure that can selectively recognize the ABR in terms of structure and shape, care should be taken not to damage this structure by removal. In Fig. 7D, we can observe how the ΔI_1_ values change when GCE is incubated in different solutions (15 M HAc, 1 M HCl, acetone, etc.) at 650 rpm with a ThermoShaker. After the most effective result was obtained with 15 M HAc, the effect of different durations between 5 and 15 min was examined (Fig. 7E). Although similar results were obtained at 10, 12, and 15 min, 10 min was chosen for time savings and repeatability.

#### Rebinding time

The rebinding phase is the phase in which the sensor selectively recognizes and binds to the ABR and determination occurs. While optimizing the time when rebinding is most effective, the determination response time is also determined. When calculating Δ*I*_2_ values, the difference between the current values obtained after removal and after rebinding is taken and plotted against the rebinding times (Fig. 7F). Here, the rebinding effect of 1.0 × 10^–11^ M ABR was tested, and considering the close results at 10, 12, and 15 min, 10 min was determined as the rebinding time.

### Evaluation of the analytical performance of the ABR/4-ABA/ZnNFs@GO/MIP/GCE sensor in standard solution

**Fig. 8:**
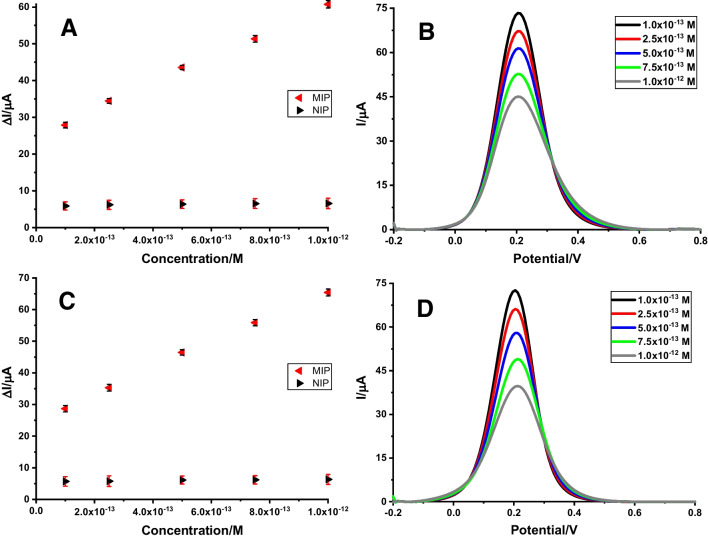
The calibration line of ABR on ABR/4-ABA/ZnNFs@GO/MIP/GCE and NIP/GCE **A** in standard solution, **C** in commercial human serum sample; DP voltammograms after rebinding of ABR (1.0 × 10^**−**13^ M – 1.0 × 10^**−**12^ M) on ABR/4-ABA/ZnNFs@GO/MIP/GCE **B** in standard solution, **D** in commercial human serum sample (the measurements were performed in 5.0 × 10^**−**13^ M [Fe(CN)_6_]^3**−**/4^.^**−**^ solution) (conditions for DPV: potential scan range, − 0.2 V to + 0.6 V; scan rate, 0.001587 V/s; step potential 8 mV; modulation amplitude 50 mV; modulation time 0.05 s, interval time 0.5 s)

The evaluation of the analytical performance of the ABR/4-ABA/ZnNFs@GO/MIP/GCE sensor in standard solution was performed using 5.0 × 10^–3^ M [Fe(CN)_6_]^3–/4–^ solution as the redox probe and utilizing the indirect measurement method. For this purpose, electrochemical measurements are not performed directly in the ABR solution at different concentrations, but the decreases in the peak current of the redox probe are calculated after binding ABR to specific cavities at different concentrations. When the acquired Δ*I*_2_ values were plotted against increasing ABR concentrations, a linear response occurred in the concentration range of 1.0 × 10^−13^ M and 1.0 × 10^−12^ M for ABR/4-ABA/ZnNFs@GO/MIP/GCE (Fig. 8A). However, this logical response could not be obtained when the measurement was performed using the same procedures with NIP, which does not contain specific cavities. This confirms the specificity of the sensor. The regression equation calculated from these values is: Δ*I*_2_ (µΑ) = 3.63 × 1013 (µA/M) × C (M) + 24.54 (*r* = 0.999). The equations in the ICH guidelines are used to calculate the limit of detection (LOD) and limit of quantification (LOQ): LOD = 3 × standard deviation / slope, and LOQ = 10 × standard deviation / slope [33]. All parameters associated with the ABR determination are presented in Table 2. The decrease in DPV currents of the redox probe after ABR binding at increasing concentrations is shown in Fig. 8B.

**Table 2:** Regression data of the calibration line on ABR/4-ABA/ZnNFs@GO/MIP/GCE

Parameter	Standard solution	Serum sample
Linearity range (M)	1.0 × 10^–13^ – 1.0 × 10^–12^	1.0 × 10^–13^ – 1.0 × 10^–12^
Slope (µA/M)	3.63 × 10^13^	4.03 × 10^13^
SE of slope (µA/M)	6.79 × 10^11^	8.57 × 10^11^
Intercept (µA)	24.54	25.38
SE of intercept (µA)	0.42	0.70
Correlation coefficient (*r*)	0.999	0.999
LOD (M)	2.18 × 10^−14^	2.36 × 10^−14^
LOQ (M)	7.28 × 10^−14^	7.86 × 10^−14^
Repeatability of response (RSD%)*	0.95	1.11
Reproducibility of response (RSD%)*	1.91	1.76

*Each value is the mean of three experiments

### Evaluation of the analytical performance of the ABR/4-ABA/ZnNFs@GO/MIP/GCE sensor in a commercial human serum sample

The commercial human serum sample prepared as described in the “Green synthesis of ZnNFs and ZnNFs@GO” section was used to test the performance of the ABR/4-ABA/ZnNFs@GO/MIP/GCE sensor in biological fluids. As in the standard solution, the linear response was obtained in the concentration range of 1.0 × 10^–13^ and 1.0 × 10^–12^ M (Fig. 8C). The calculated LOD and LOQ values and other relevant parameters for the ABR determination in the serum sample are summarized in Table 2. The decrease in DPV currents of the redox probe after ABR binding at increasing concentrations is shown in Fig. 8D. The ABR standard solution was then spiked into the serum samples at two different known concentrations to demonstrate the accuracy of the ABR/4-ABA/ZnNFs@GO/MIP/GCE sensor. Table 3 shows that the recovery and associated RSD% values were good and the performance of the sensor was verified.

**Table 3:** Recovery experiment results for the samples of commercial human serum

Parameter	Serum sample no. 1	Serum sample no. 2
Sample concentration (M)	2.50 × 10^−13^	5.00 × 10^−13^
Spiked amount (M)	5.00 × 10^−13^	2.50 × 10^−13^
Found amount (M)*	7.48 × 10^−13^	7.53 × 10^−13^
Average recovery (%)*	101.04	101.56
RSD% of recovery	1.93	1.94
Bias%	+ 1.04	+ 1.56

*Each value is the mean of five experiments

### Effect of interfering substances

Since the ABR/4-ABA/ZnNFs@GO@MIP/GCE sensor can be applied to biological samples for the selective and sensitive determination of ABR, it is also necessary to evaluate substances that may be present in biological fluids and cause interference. In this context, interfering substances were selected and their effect on the determination of ABR by the ABR/4-ABA/ZnNFs@GO@MIP/GCE sensor was investigated when the target molecule was present at a concentration 10 times higher than the ABR. According to the calculated recovery% values, which are between 99.14% and 102.48% (Table 4), the ABR/4-ABA/ZnNFs@GO@MIP/GCE sensor can sensitively and selectively determine ABR without observing the interfering effect of these substances.

**Table 4:** Interference study results of ABR/4-ABA/ZnNFs@GO@MIP/GCE sensor for the determination of ABR

Interfering substances (IS)*	Recovery of ABR (%)	RSD (%)
K^+^	101.17	1.07
NO_3_^−^	101.17	1.07
Mg^2+^	99.14	1.78
Cl^−^	99.14	1.78
Na^+^	99.88	1.67
SO_4_^2−^	99.88	1.67
Dopamine	101.92	1.32
Uric acid	101.99	1.50
Ascorbic acid	102.37	1.38
Paracetamol	102.48	1.32

*Molar ratio of ABR/IS is 1:10

### Imprinting factor study

Imprinting factor (IF) calculation studies were performed to express the selectivity and ability to specifically recognize the target molecule, which are the most critical features of the sensor developed for ABR determination and the greatest advantage of the MIP technique. The MIP design creates selective cavities that complement the chemical structure and functional groups of the target molecule. For this reason, ibrutinib, ruxolitinib, tofacitinib, zonisamide, and acetazolamide, molecules structurally similar to ABR, were selected for the IF study. To determine the affinity of the ABR/4-ABA/ZnNFs@GO@MIP/GCE sensor for each compound and whether these compounds bind to the cavities in MIP, Δ*I*_2_ values for MIP and NIP were calculated and relative IF (IF′) values were found with their ratios to each other. The results obtained are shown in Table 5 and the fact that the IF′ values are greater than 1 emphasizes the selectivity of the sensor towards ABR.

**Table 5:**
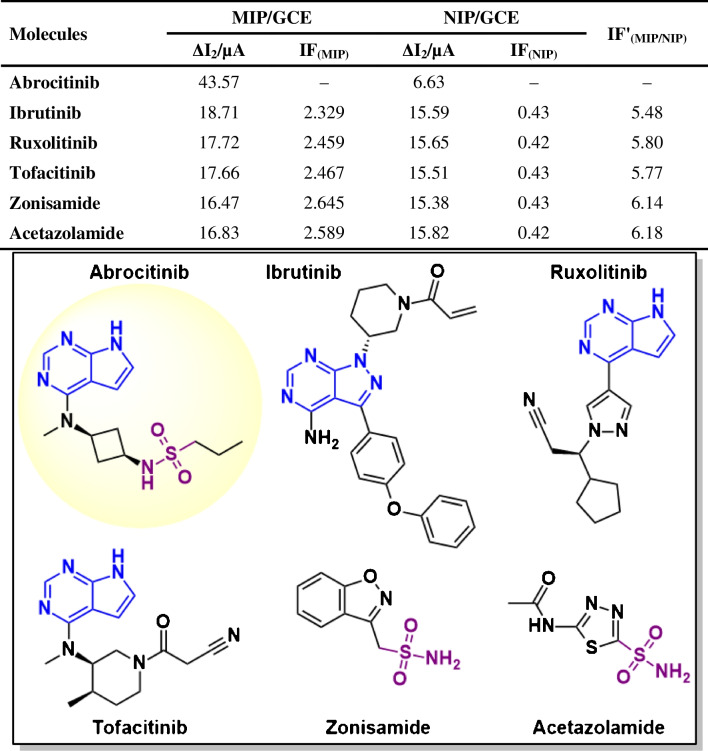
Selectivity study results for ABR/4-ABA/ZnNFs@GO@MIP/GCE sensor on ABR determination

### Stability and reusability testing

The ABR/4-ABA/ZnNFs@GO@MIP/GCE sensor was stored in a desiccator at room temperature and used for stability measurements for 10 days. The designed sensor showed a performance of 97.44% in the first 3 days of storage, 95.28% in 5 days of storage, 90.66% in 7 days of storage, and 86.92% in 10 days of storage. As a result, the stability of the ABR/4-ABA/ZnNFs@GO@MIP/GCE sensor was found to be 5 days. Furthermore, to evaluate the reusability of the constructed sensor, 10 repeatable results were obtained after rebinding.

## Conclusion

In the NF structure, *Alkanna cappadocica* root extract was used as the organic compound, and zinc acetate was used as the inorganic compound. It is thought that compounds in the naphthoquinone structure found in the root extract of the plant participate in nanoflower formation. Also, zinc nanoflower was synthesized using *A. cappadocica* roots for the first time. Thus, zinc phosphate nanoflower structures were synthesized in PBS buffer environment without the use of chemical agents. Studies have shown that these structures have a high surface area/volume ratio and high porosity. Because of these advantages and because it is of herbal origin, this study designed the green synthesis ZnNFs@GO-assisted MIP-based sensor (ABR/4 ABA/ZnNFs@GO@MIP/GCE) and described the first electrochemical application of ABR determination with MIP-based sensor. It was also successfully applied to serum samples, which had advantages such as high selectivity, sensitivity, affordable cost, simple procedure, and stability. While the surface properties of ABR/4-ABA/ZnNFs@GO@MIP/GCE were characterized in detail both electrochemically and morphologically, CV, EIS, SEM, FTIR, and XRD methods were applied. The sensor, which is linear in the concentration range of 1.0 × 10^–13^ and 1.0 × 10^–12^ M in both standard solution and biological media, is also very advantageous in terms of LOD and LOQ values. In addition, the accuracy has been demonstrated by recovery studies. The specificity and selectivity of the ABR/4-ABA/ZnNFs@GO@MIP/GCE sensor, which is the most prominent feature, have been confirmed by IF calculations using drugs with structural motifs similar to ABR and by interference studies using the molecules and ions present in biological fluids. As a result, the ABR/4-ABA/ZnNFs@GO@MIP/GCE sensor is recommended as an advantageous sensor approach for the determination of ABR, which has become a novel and highly effective option in the treatment of atopic dermatitis.

## Data Availability

The datasets generated during and/or analyzed during the current study are available from the corresponding author upon reasonable request.

## References

[1] Gao Q, Zhao Y, Zhang J (2023). Efficacy and safety of abrocitinib and upadacitinib versus dupilumab in adults with moderate-to-severe atopic dermatitis: a systematic review and meta-analysis. Heliyon.

[2] Grajales DB, Sewdat N, Leo R, Kar S (2023). Unveiling abrocitinib: a thorough examination of the 2022 USFDA-approved treatment for atopic dermatitis (AD). Med Drug Discov.

[3] Alexis AF, Silverberg JI, Rice ZP et al (2023) Abrocitinib efficacy and safety in moderate-to-severe atopic dermatitis by race, ethnicity, and Fitzpatrick skin type. Ann Allergy, Asthma Immunol 132:383–389. 10.1016/j.anai.2023.11.002 10.1016/j.anai.2023.11.002 37949351

[4] Bauman JN, Doran AC, King-Ahmad A (2022). The pharmacokinetics, metabolism, and clearance mechanisms of abrocitinib, a selective Janus kinase inhibitor, in humans. Drug Metab Dispos.

[5] McLornan DP, Pope JE, Gotlib J, Harrison CN (2021). Current and future status of JAK inhibitors. The Lancet.

[6] Tachet J, Versace F, Mercier T (2023). Development and validation of a multiplex HPLC-MS/MS assay for the monitoring of JAK inhibitors in patient plasma. J Chromatogr B Analyt Technol Biomed Life Sci.

[7] Wang X, Dowty ME, Wouters A (2022). Assessment of the effects of inhibition or induction of CYP2C19 and CYP2C9 enzymes, or inhibition of OAT3, on the pharmacokinetics of abrocitinib and its metabolites in healthy individuals. Eur J Drug Metab Pharmacokinet.

[8] Wojciechowski J, Malhotra BK, Wang X (2022). Population pharmacokinetics of abrocitinib in healthy individuals and patients with psoriasis or atopic dermatitis. Clin Pharmacokinet.

[9] Cetinkaya A, Kaya SI, Alahmad W (2023). Designing an electrochemical sensor based on ZnO nanoparticle-supported molecularly imprinted polymer for ultra-sensitive and selective detection of sorafenib. Anal Chim Acta.

[10] Chen L, Wang X, Lu W (2016). Molecular imprinting: perspectives and applications. Chem Soc Rev.

[11] Cetinkaya A, Unal MA, Nazır H (2023). Two different molecularly imprinted polymeric coating techniques for creating sensitive and selective electrochemical sensors for the detection of Ribavirin. Sens Actuators B Chem.

[12] Budak F, Cetinkaya A, Kaya SI et al (2023) Investigations on the electrochemical behavior of sunitinib and metabolites N-desethyl-sunitinib and sunitinib-N-oxide and its selective determination using molecularly imprinted polymer-based sensor. Electrochim Acta 472:143434. 10.1016/j.electacta.2023.143434

[13] Kumar V, Yadav SK (2009) Plant-mediated synthesis of silver and gold nanoparticles and their applications. J Chem Technol Biotechnol 84:151–157. 10.1002/jctb.2023

[14] Badgujar HF, Kumar U (2021) Green approach towards morphology-controlled synthesis of zein-functionalized TiO2 nanoparticles for cosmeceutical application. Eur J Pharm Sci 167:106010. 10.1016/j.ejps.2021.106010 10.1016/j.ejps.2021.106010 34537374

[15] Shcharbin D, Halets-Bui I, Abashkin V et al (2019) Hybrid metal-organic nanoflowers and their application in biotechnology and medicine. Colloids Surf B Biointerfaces 182:110354. 10.1016/j.colsurfb.2019.110354 10.1016/j.colsurfb.2019.110354 31325775

[16] Liu Y, Ji X, He Z (2019). Organic-inorganic nanoflowers: From design strategy to biomedical applications. Nanoscale.

[17] Zhang L, Wang Y, Wang Y et al (2023) Electrochemical H2O2 sensor based on a Au nanoflower-graphene composite for anticancer drug evaluation. Talanta 261:124600. 10.1016/j.talanta.2023.124600 10.1016/j.talanta.2023.124600 37216890

[18] Shende P, Kasture P, Gaud RS (2018). Nanoflowers: the future trend of nanotechnology for multi-applications. Artif Cells Nanomed Biotechnol.

[19] Zhang B, Lu L, Hu Q, et al (2014) ZnO nanoflower-based photoelectrochemical DNAzyme sensor for the detection of Pb2+. Biosens Bioelectron 56:243–249. 10.1016/j.bios.2014.01.026 10.1016/j.bios.2014.01.026 24508815

[20] Fang B, Zhang C, Zhang W, Wang G (2009) A novel hydrazine electrochemical sensor based on a carbon nanotube-wired ZnO nanoflower-modified electrode. Electrochim Acta 55:178–182. 10.1016/j.electacta.2009.08.036

[21] Ge J, Lei J, Zare RN (2012) Protein-inorganic hybrid nanoflowers. Nat Nanotechnol 7:428–432. 10.1038/nnano.2012.80 10.1038/nnano.2012.80 22659609

[22] Özdemir N, Altinkaynak C, Türk M et al (2022) Amino acid-metal phosphate hybrid nanoflowers (AaHNFs): their preparation, characterization and anti-oxidant capacities. Polym Bull 79:9697–9716. 10.1007/s00289-021-03973-7

[23] Caires AJ, Alves DCB, Fantini C et al (2015) One-pot in situ photochemical synthesis of graphene oxide/gold nanorod nanocomposites for surface-enhanced Raman spectroscopy. RSC Adv 5:46552–46557. 10.1039/c4ra17207h

[24] Zheng W, Fan H, Wang L, Jin Z (2015) Oxidative self-polymerization of dopamine in an acidic environment. Langmuir 31:11671–11677. 10.1021/acs.langmuir.5b02757 10.1021/acs.langmuir.5b02757 26442969

[25] Ildiz N, Korkmaz F, Yusufbeyoğlu S et al (2021) Silver nanoparticles biosynthesized from Vaccinium myrtillus L. against multiple antibiotic resistance and biofilm forming escherichia coli and pseudomonas aeruginosa. Indian J Pharm Educ Res 55:220–s224. 10.5530/ijper.55.1s.53

[26] Zhao K, Feng L, Lin H (2014). Adsorption and photocatalytic degradation of methyl orange imprinted composite membranes using TiO2/calcium alginate hydrogel as matrix. Catal Today.

[27] Guo L, Ma X, Xie X (2019). Preparation of dual-dummy-template molecularly imprinted polymers coated magnetic graphene oxide for separation and enrichment of phthalate esters in water. Chem Eng J.

[28] Baldemir A, Köse NB, Ildiz N (2017). Synthesis and characterization of green tea (Camellia sinensis (L.) Kuntze) extract and its major components-based nanoflowers: a new strategy to enhance antimicrobial activity. RSC Adv.

[29] Baldemir Kılıç A, Ildız N, Yusufbeyoğlu S, Öçsoy İ (2023) Nanoflower synthesis formed at different pH based on Crocus sativus L. (Croci stigma, saffron) extract and its major components: a new approach for enhancing antioxidant, antimicrobial and catalytic activities. Inorg Nano-Metal Chem. 10.1080/24701556.2023.2240757

[30] Bor E, Koca Caliskan U, Anlas C (2022). Synthesis of Persea americana extract based hybrid nanoflowers as a new strategy to enhance hyaluronidase and gelatinase inhibitory activity and the evaluation of their toxicity potential. Inorg Nano-Metal Chem.

[31] Yilmaz BS, Bekci H, Altiparmak A (2024). Determination of anticancer activity and biosynthesis of Cu, Zn, and Co hybrid nanoflowers with Tribulus terrestris L. extract. Process Biochem.

[32] Wackerlig J, Lieberzeit PA (2015). Molecularly imprinted polymer nanoparticles in chemical sensing - synthesis, characterisation and application. Sens Actuators B Chem.

[33] European Medicines Agency (1995) Validation of analytical procedures: text and methodology. international conference on harmonisation (ICH) Guideline ICH Topic Q2(R1)

